# Novel steerable-tip catheter successfully used for cannulation of a steeply angled pancreaticojejunostomy anastomotic stenosis

**DOI:** 10.1055/a-2491-4593

**Published:** 2025-01-14

**Authors:** Haruka Toyonaga, Takuya Takayama, Tatsuya Nakagawa, Masataka Kano, Masahiro Orino, Hironao Matsumoto, Masaaki Shimatani

**Affiliations:** 1 50196 Gastroenterology and Hepatology, Kansai Medical University Medical Center, Moriguchi, Japan

Pancreaticojejunostomy anastomotic stenosis following pancreaticoduodenectomy is one of the most challenging issues an endoscopist can face. Its management often requires balloon enteroscopy-assisted endoscopic retrograde cholangiopancreatography (BE-ERCP); however, several obstacles can impede the successful completion of the procedure. These include difficulty in accessing the anastomosis owing to adhesions, challenges in identifying the anastomosis, and difficulty in selective pancreatic duct cannulation due to the anastomotic stenosis in combination with the steep angulation between the afferent limb and the pancreatic duct that is inherent to this surgical reconstruction ( Fig. 1 ) 1 2 . Various techniques have been reported to address these challenges 3 , but achieving successful cannulation of the pancreaticojejunostomy remains difficult, occasionally necessitating endoscopic ultrasonography (EUS)-guided interventions. Recently, a newly developed steerable-tip catheter (KC226; Zeon Medical, Tokyo, Japan), with the tip composed of expanded polytetrafluoroethylene (ePTFE) that has a maximum bending angle of ±90° 4 5 , has become available, offering effective cannulation for pancreaticojejunostomy anastomotic stenosis with severe angulation ( Fig. 2 ).

**Fig. 1:**
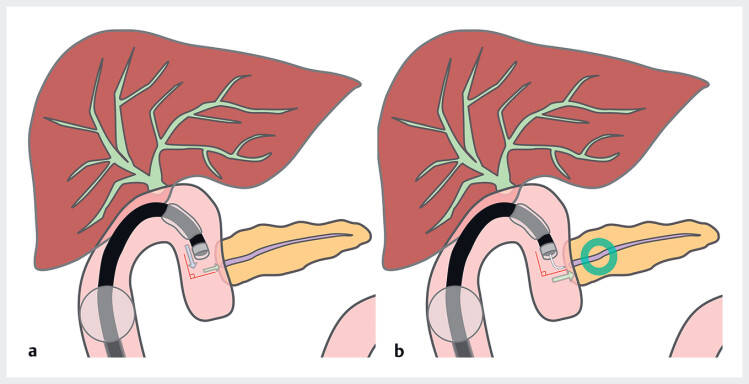
Schematic of balloon enteroscopy-assisted endoscopic retrograde cholangiopancreatography for pancreaticojejunostomy anastomotic stenosis following pancreaticoduodenectomy showing: **a** the steep angle formed by the afferent limb and the pancreatic duct; **b** how the steerable-tip catheter, with a tip that can bend to an angle of 90°, facilitates alignment with the pancreatic duct axis.

**Fig. 2:**
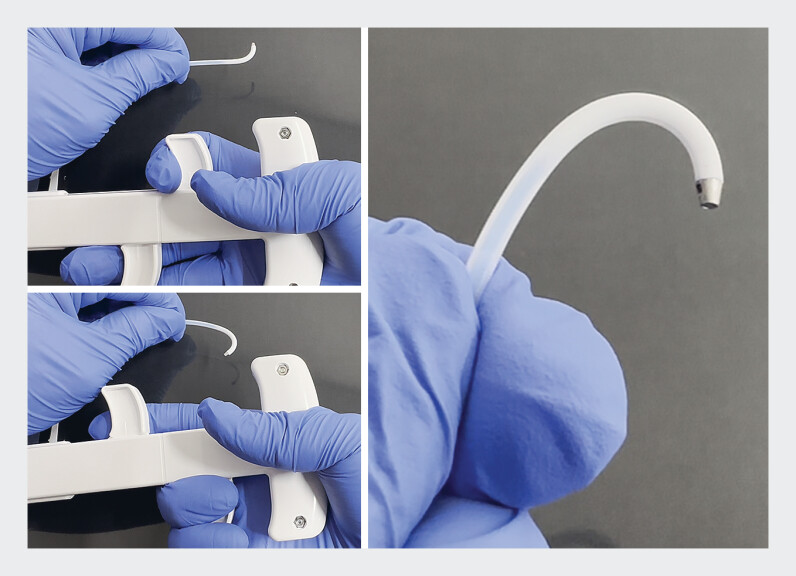
Photographs of the newly developed steerable-tip catheter (KC226; Zeon Medical, Tokyo, Japan), which has a tip composed of expanded polytetrafluoroethylene (ePTFE) that can bend to a maximum angle of ±90°.

A woman in her eighties who had undergone pancreaticoduodenectomy for distal bile duct cancer, presented with a pancreaticojejunostomy anastomotic stenosis. During BE-ERCP, the anastomosis was successfully reached and identified endoscopically ( Fig. 3 ); however, it was not possible to cannulate the pancreaticojejunostomy anastomotic stenosis using a conventional catheter because of the tight stricture and sharp anastomotic angle ( Fig. 4 ). Using the new steerable-tip catheter, we were able to align with the axis of the pancreatic duct and achieve selective cannulation ( Fig. 5 ). The stricture was dilated with a dilator and a stent was placed, concluding the procedure ( Video 1 ).

**Fig. 3:**
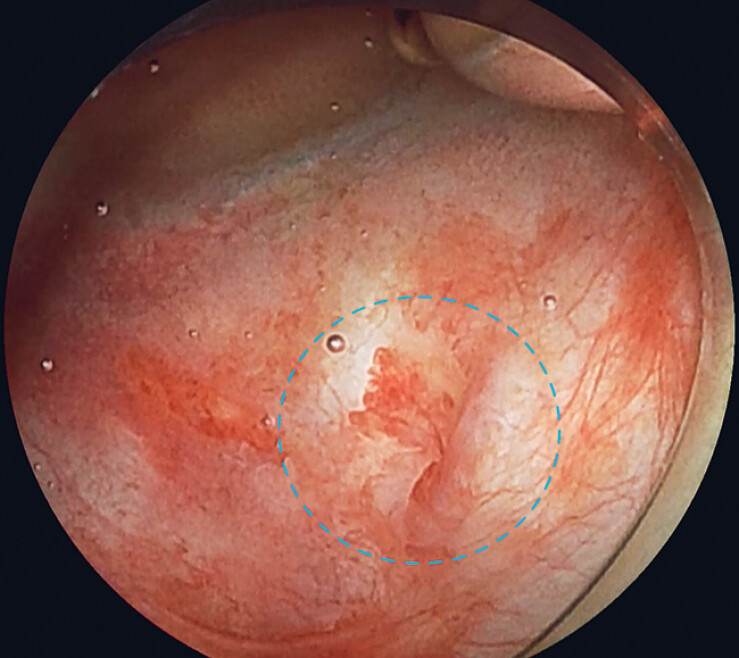
Endoscopic image of the pancreaticojejunostomy anastomosis site (blue dashed circle).

**Fig. 4:**
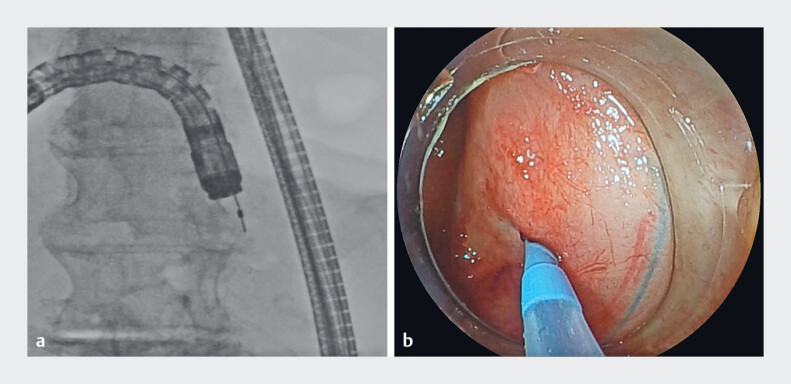
Failure to cannulate the pancreaticojejunostomy anastomotic stenosis using a conventional catheter because of a tight stricture and steep anastomotic angle is shown on: **a** fluoroscopic image; **b** endoscopic image.

**Fig. 5:**
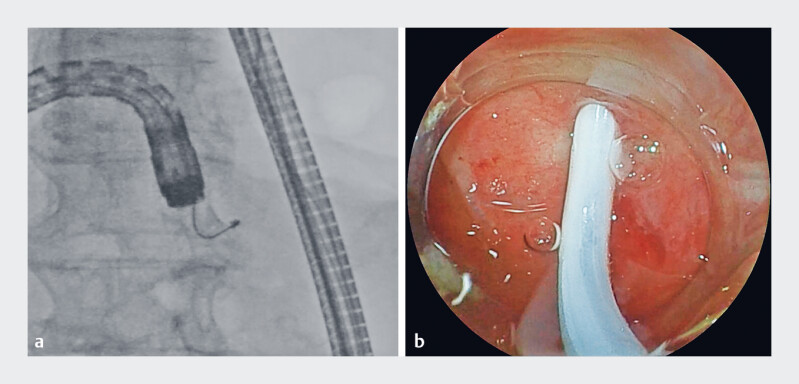
A successful approach to the steeply angled pancreaticojejunostomy anastomotic stenosis by bending the tip of the steerable catheter to an angle of 90° is shown on: **a** fluoroscopic image; **b** endoscopic image.

The novel steerable-tip catheter is used to facilitate cannulation in a steeply angled pancreaticojejunostomy anastomotic stenosis during balloon enteroscopy-assisted endoscopic retrograde cholangiopancreatography.Video 1

This case demonstrates the utility of the steerable-tip catheter for overcoming the challenges of cannulation in steeply angled pancreaticojejunostomy anastomotic stenosis during BE-ERCP.

Endoscopy_UCTN_Code_TTT_1AR_2AG

## Notes

Endoscopy E-Videos https://eref.thieme.de/e-videos
*E-Videos* is an open access online section of the journal *Endoscopy* , reporting on interesting cases and new techniques in gastroenterological endoscopy. All papers include a high-quality video and are published with a Creative Commons CC-BY license. Endoscopy E-Videos qualify for HINARI discounts and waivers and eligibility is automatically checked during the submission process. We grant 100% waivers to articles whose corresponding authors are based in Group A countries and 50% waivers to those who are based in Group B countries as classified by Research4Life (see: https://www.research4life.org/access/eligibility/ ). This section has its own submission website at https://mc.manuscriptcentral.com/e-videos .
